# Translational Barriers and Optimization Strategies for Remote Ischemic Conditioning to Enhance Stroke Cerebroprotection

**DOI:** 10.3390/biom16040568

**Published:** 2026-04-11

**Authors:** Xin Zhang, Jiaxin An, Xiaofeng Guo, Jiayu Li, Ruimin Wang

**Affiliations:** 1 Institute of Neurobiology, School of Public Health, North China University of Science and Technology, Tangshan 063210, China; xwb@ncst.edu.cn (X.Z.); ajx0101@163.com (J.A.); guoxfeng03@163.com (X.G.); 2 International Science & Technology Cooperation Base of Geriatric Medicine, Tangshan 063210, China; 3 School Basic Medical Science, North China University of Science and Technology, Tangshan 063210, China; ljy17695003274@163.com

**Keywords:** stroke, remote ischemic conditioning, RIC resistance, signal transmission, cerebroprotection

## Abstract

Remote ischemic conditioning (RIC) is an endogenous strategy that mitigates cerebral injury in preclinical stroke models. However, its bench-to-bedside translation is frequently hindered by complex patient environments that induce RIC resistance and limit its neuroprotective efficacy. To bridge this translational gap, this review systematically examines the extrinsic pathophysiological and pharmacological barriers to RIC. We categorize RIC resistance into three mechanism-driven phenotypes. Impaired signal initiation (Type I) is often linked to diabetic sensorimotor polyneuropathy and the reactive oxygen species-scavenging effects of propofol. Signal transmission blockade (Type II) is associated with specific P2Y12 inhibitors and smoking-induced endothelial dysfunction. Furthermore, effector desensitization (Type III) involves target-organ unresponsiveness exacerbated by aging, chronic hyperglycemia, and postmenopausal estrogen depletion. To address these barriers, potential phenotype-specific optimization strategies are discussed. Ultimately, transitioning from generalized empirical protocols to mechanism-based precision strategies may help bypass RIC resistance in clinical settings and enhance stroke cerebroprotection.

## 1. Introduction

Ischemic stroke remains a leading cause of global mortality and long-term disability, underscoring the urgent clinical need for effective cerebroprotective therapies [1,2]. In recent years, Remote Ischemic Conditioning (RIC) has gained substantial traction as a promising, non-invasive intervention and is being increasingly investigated in clinical treatment paradigms for acute ischemic stroke [3,4]. Originally described as remote myocardial preconditioning by Przyklenk et al. in 1993 [5], RIC is typically administered via repeated cycles of transient limb ischemia and reperfusion using a standard blood pressure cuff applied to an upper or lower extremity, triggering systemic organ-protective signaling cascades [6]. In preclinical animal models, RIC has been shown to limit infarct size [7,8,9,10], improve neurological function scores [11,12], and preserve mitochondrial function [13,14]. However, translating the stable and reproducible protective effects observed in experimental animals to clinical practice has encountered significant challenges [15,16]. Although some clinical trials, including RICAMIS [17] and REMOTE-CAT [18], have demonstrated the efficacy of RIC in improving post-intervention functional prognosis [19,20], large-scale randomized controlled trials (RCTs) such as RESIST [21] and SERIC-IVT [22] have failed to confirm the efficacy of RIC in improving long-term clinical outcomes.

This translational gap necessitates a critical re-evaluation of RIC cerebroprotective efficacy within contemporary clinical contexts. Today, acute stroke management relies heavily on primary recanalization approaches, particularly intravenous thrombolysis and mechanical thrombectomy [23,24]. Consequently, cerebroprotective strategies like RIC are most valuable when evaluated as adjunctive treatments designed to extend the therapeutic time window and mitigate subsequent ischemia–reperfusion injury rather than as isolated therapies.

A major driver of the aforementioned translational gap is the stark baseline difference between clinical and preclinical study subjects. Stroke patients receiving advanced reperfusion therapies have heterogeneous profiles marked by advanced age, diabetes, hypertension, and complex drug regimens including anesthesia and antiplatelet therapy. These profiles differ sharply from the young, genetically homogeneous animal models used in laboratory settings. RIC is not a localized phenomenon but a complex systemic reflex. The cerebroprotective signal generated in the limb must traverse a systemic neuro-humoral network to reach the ischemic brain. Consequently, clinical stroke cerebroprotection is fundamentally dictated by the integrity of this systemic transit. While variability in the physical parameters of RIC protocols (e.g., cuff pressure, cycle duration) contributes to inconsistent outcomes, standardizing these variables has not resolved the translational failures in large RCTs. This indicates that procedural mechanical parameters are not the sole barrier to translation. Therefore, this review shifts focus to the clinical pathophysiological and pharmacological factors that directly impair the systemic signaling cascades initiated by RIC.

The protective pathways activated by RIC are highly susceptible to interference from the aforementioned clinical comorbidities and routine medications. We formally define this translational barrier as RIC resistance: the failure of RIC to elicit its expected biological protective effects and improve clinical outcomes, resulting from the impairment of the RIC-triggered signaling cascade. The objective of this review is to examine how clinical variables disrupt this network and to propose strategies for optimizing cerebroprotection. Specifically, this review is structured as follows: (1) the mechanisms and translational challenges of RIC in stroke cerebroprotection; (2) the pathophysiological and pharmacological barriers to RIC efficacy; (3) the mechanism-driven phenotypic stratification of RIC resistance (Types I, II, and III); (4) phenotype-specific precision RIC strategies; and (5) current limitations and future translational perspectives.

## 2. Mechanisms and Translational Challenges of RIC in Stroke Cerebroprotection

### 2.1. The Biological Progenitors and Shared Principles of RIC

To elucidate the systemic cascade of RIC, it must first be situated within the broader conceptual framework of the conditioning phenomenon. Tracing its biological lineage back to classical local ischemic preconditioning, RIC represents a systemic evolution of this protective paradigm [25,26]. Whether administered as preconditioning, perconditioning, or postconditioning, all these variants rely on a highly conserved biological principle [27]. This principle states that a sub-lethal stressor triggers an endogenous protective phenotype. A universal and essential trigger in this shared mechanism is the transient, physiological burst of signaling reactive oxygen species (ROS) [28,29,30]. These signaling radicals act as critical secondary messengers to activate conserved survival networks, including the reperfusion injury salvage kinase (RISK) and survivor activating factor enhancement (SAFE) pathways [30,31,32]. While RIC inherits these fundamental intracellular pathways, it possesses unique mechanistic requirements, primarily the necessity of a complex neurohumoral bridge to transmit protective signals from the peripheral limb to distant organs, with the ischemic brain as the ultimate target [33].

### 2.2. The Three-Step Systemic Cascade of RIC

**Figure 1:**
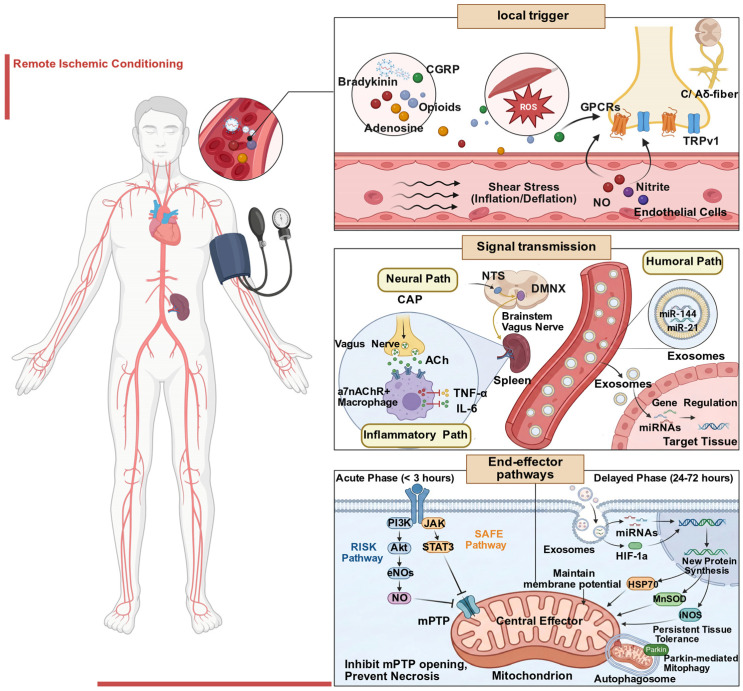
The mechanism of Remote Ischemic Conditioning (RIC)-mediated multi-organ protection. (1) Local trigger: Transient limb ischemia–reperfusion induces the release of local autacoids, reactive oxygen species (ROS), and shear stress-mediated nitric oxide (NO). These redox and chemical signals activate sensory afferents (C/Aδ-fibers) via G protein-coupled receptors (GPCRs) and transient receptor potential cation channel subfamily V member 1 (TRPV1) channels. (2) Signal transmission: The protective signal propagates systemically through neural, inflammatory, and humoral axes. The vagal reflex suppresses splenic proinflammatory cytokines (tumor necrosis factor-alpha, TNF-α; interleukin-6, IL-6) via acetylcholine (ACh) release binding to alpha-7 nicotinic acetylcholine receptor (α7nAChR)-positive macrophages, while circulating exosomes deliver gene-regulatory microRNAs(e.g., miR-144, miR-21) to distant tissues. (3) End-effector pathways: Mitochondria serve as the central protective hub. In the acute phase (<3 h), reperfusion injury salvage kinase (RISK) pathway and survivor activating factor enhancement (SAFE)pathway activation rapidly inhibits mitochondrial permeability transition pore (mPTP) opening to prevent necrosis. In the delayed phase (24–72 h), transcriptional regulation (e.g., hypoxia-inducible factor-1-alpha, HIF-1α) drives the de novo synthesis of antioxidant enzymes (manganese superoxide dismutase, MnSOD; inducible nitric oxide synthase, iNOS) and chaperones (heat shock protein 70, HSP70), alongside enhanced Parkin-mediated mitophagy, conferring persistent ischemic tolerance.

The fundamental mechanism of RIC involves applying short-term, sub-lethal ischemia–reperfusion stimuli to the distal limbs or tissues, which induces a systemic protective response, thereby mitigating reperfusion injury caused by subsequent ischemic stroke [34]. To orchestrate this cross-organ protection, the RIC-induced cascade structurally relies on three interdependent steps: local signal initiation, signal transmission, and end-effector pathway activation in the target organ (Figure 1) [35,36]. Pathological or pharmacological interference at any of these three steps will lead to corresponding phenotypic RIC resistance, which is the theoretical basis of the classification system proposed in this review. Notably, the specific molecular cascades of RIC depend heavily on the timing of the intervention. Prophylactic preconditioning prior to elective surgery may engage slightly different pathways compared to therapeutic postconditioning utilized during an acute ischemic stroke. However, this review primarily focuses on how extrinsic patient-specific factors compromise these shared protective networks, regardless of the conditioning timeline.

#### 2.2.1. Local Signal Initiation

The initiation of RIC-mediated protective signaling involves a dual-trigger mechanism. First, limb ischemia–reperfusion induces a rapid shift in local metabolic patterns. The reoxygenation phase triggers the production of signaling ROS, which work together with a mixture of autacoids, including adenosine, bradykinin, opioids, and the neuropeptide calcitonin gene-related peptide (CGRP) [37,38]. While these autacoids activate G protein-coupled receptors (GPCRs), signaling ROS likely modulate redox-sensitive targets directly. Second, physical stimulation of the blood vessel wall plays an equally critical role. Repeated inflation/deflation cycles lead to significant fluctuations in shear stress, activating vascular endothelial cells to release nitric oxide (NO) and nitrite [39]. NO acts as a vasodilator and directly regulates mitochondrial function through S-nitrosylation. These two types of signals act together on the peripheral sensory nerve endings of C fibers and Aδ fibers, converting chemical and physical signals into afferent nerve impulses via transient receptor potential (TRP) channels, particularly TRP vanilloid 1 [40]. The observation that femoral nerve transection or capsaicin-induced neurotransmitter depletion markedly attenuates the protective effect of RIC confirms that an intact neural pathway is a critical prerequisite for the initiation of RIC’s protective signaling [41,42]. Impairment of this local trigger step is the mechanistic basis of Type I (Impaired Signal Initiation) RIC resistance.

#### 2.2.2. Signal Transmission

The afferent signal projects from the spinal cord to the nucleus tractus solitarius (NTS). The NTS then activates the dorsal motor nucleus of the vagus nerve (VN), initiating a characteristic mode of neurohumoral interdependent transmission [43]. The efferent vagal fibers modulate the spleen via the cholinergic anti-inflammatory pathway (CAP) [44,45]. Specifically, efferent vagal signals are relayed in the celiac ganglion to activate the splenic nerve, which releases norepinephrine to stimulate choline acetyltransferase (ChAT)-positive T cells. These T cells synthesize and release acetylcholine, which subsequently activates splenic **alpha-7 nicotinic acetylcholine receptor-positive (α7nAChR+)** macrophages, inhibiting the release of systemic pro-inflammatory factors, such as tumor necrosis factor-alpha (TNF-α) and interleukin-6 (IL-6), and reprogramming the systemic immune environment into an anti-inflammatory and repair-promoting state [46,47,48]. Furthermore, RIC-induced extracellular vesicles (EVs), particularly exosomes, serve as important vehicles for cross-organ systemic signal transmission. Studies confirm that RIC exosomes encapsulate specific microRNAs (miRNAs), including miR-144 [49] and miR-21 [50]. These encapsulated miRNAs are subsequently delivered to the ischemic myocardium or cross the blood–brain barrier (BBB) to reach brain tissue [51]. This vesicle transport mechanism explains why RIC can produce long-lasting changes in gene expression. Interruption of this systemic signal propagation step is the mechanistic basis of Type II (Signal Transmission Blockade) RIC resistance.

#### 2.2.3. End-Effector Pathways

The protective efficacy of RIC exhibits a distinct biphasic temporal pattern [52]. As extensively demonstrated in classical local conditioning models, the acute phase (within three hours) relies on the rapid activation of cell membrane receptors that trigger intracellular kinase cascades, specifically the RISK (PI3K/Akt/eNOS) and SAFE (JAK/STAT3) pathways. These cascades acutely inhibit the opening of the mitochondrial permeability transition pore (mPTP) to prevent immediate cell necrosis [53]. The delayed phase (24–72 h) depends on widespread gene transcription and de novo protein synthesis. During this phase, exosomes deliver miRNAs and nuclear transcription factors, such as hypoxia-inducible factor 1 alpha (HIF-1α), to induce the expression of key defensive effectors, including heat shock protein 70 (HSP70), manganese superoxide dismutase (MnSOD), and inducible nitric oxide synthase (iNOS) [54,55,56]. While mitochondria are critical targets for maintaining energy homeostasis and clearing damaged organelles via mitophagy [57,58], gene-dependent cytosolic processes are equally vital for conferring persistent tissue tolerance and cerebroprotection. Desensitization or dysfunction of these end-effector networks in the brain forms the mechanistic basis for Type III (Effector Desensitization) RIC resistance.

The heterogeneity in the clinical efficacy of RIC arises predominantly from the inherent vulnerability of its multi-organ, three-step signal transmission cascade. RIC-derived protective signals typically propagate through peripheral nerves, the spleen, and the systemic circulation before crossing the BBB, and the structural and functional integrity of this cascade is highly susceptible to disruption. In real-world stroke management, complex clinical conditions, including advanced age, systemic comorbidities such as diabetes mellitus, and concomitant administration of anesthetics or thrombolytic agents, have been shown to frequently impair one or more links of this cascade. These impairments may ultimately manifest as the clinical phenotype of RIC resistance.

## 3. Pathophysiological and Pharmacological Barriers to RIC Neuroprotective Efficacy

### 3.1. Diabetes-Induced Impairment of the Neural and Molecular Mechanisms of RIC-Mediated Cerebroprotection

Diabetes is the most common comorbidity in patients with ischemic stroke. It is a critical factor that undermines the neuroprotective effects of RIC, acting as a major translational barrier. Studies indicate that RIC is less effective in animal models of diabetes and in clinical populations [53,59,60,61]. This RIC resistance does not arise from a single factor, but rather from the convergence of structural and molecular pathologies. Specifically, diabetic neuropathy impairs the afferent neural pathways essential for RIC signal transmission, and metabolic disturbances stemming from hyperglycemia and insulin resistance obstruct the activation of intracellular protective signaling cascades. Together, these aberrations cause a systemic failure of endogenous protection.

At the level of neural conduction, the protective effect of RIC relies on an intact neuro-humoral reflex arc. According to the neural hypothesis, limb ischemia triggers the local release of key signaling molecules, such as adenosine, bradykinin, and CGRP, which activate C- and Aδ-fiber-dominant sensory afferent nerves. These signals then propagate via the spinal dorsal root ganglion to the central nervous system. There, they exert effects on target organs, such as the ischemic brain, through efferent pathways, like the vagus nerve. However, the extensive axonal degeneration and demyelination characteristic of diabetic sensorimotor polyneuropathy (DSPN) fundamentally disrupt this anatomical basis of signal transmission. Clinical studies have confirmed that the cardioprotective effect of RIC is significantly impaired in type 2 diabetic patients with DSPN [62]. Animal experiments corroborate this finding by demonstrating that chemical nerve blockade or surgical denervation mimics the loss of RIC efficacy. However, it is crucial to emphasize that while an intact peripheral neural network is universally required for RIC signal initiation, cardioprotection and cerebroprotection are distinct phenomena that share only partially overlapping mechanisms. Further investigation in cardiovascular models reveals that this neural dysfunction is underpinned by specific molecular pathologies involving the abnormal accumulation of p35 protein and excessive activation of cyclin-dependent kinase 5 (CDK5). This leads to the formation of a p35/CDK5 complex that inhibits axon regeneration via a downstream cascade [63,64]. Consequently, the impairment of RIC stimulus transmission in diabetic patients is not merely structural but is also sustained by specific molecular abnormalities. Simply increasing the intensity of external ischemic stimulation is therefore often insufficient to fully restore protection through these compromised pathways. Whether this specificp35/CDK5-mediated axonal impairment identically dictates the loss of RIC’s cerebroprotective axis requires direct verification in preclinical cerebral ischemia models.

At the cellular level, the end-organ protective effects of RIC depend on the RISK pathway, particularly the activation of the PI3K/Akt/eNOS signaling axis [65,66]. However, the hyperglycemic environment impedes this pathway through glucotoxicity-induced oxidative stress and aberrant post-translational modifications [66,67]. First, hyperglycemia increases flux through the hexosamine biosynthesis pathway (HBP). This results in the excessive production of UDP-GlcNAc, which is subsequently catalyzed by *O*-linked β-*N*-acetylglucosamine (*O*-GlcNAc) transferase (OGT) to generate extensive *O*-GlcNAc modifications on proteins [67,68]. Importantly, the serine 1177 (Ser1177) site, essential for eNOS activation, is targeted by both Akt phosphorylation and *O*-GlcNAc modification. Excessive *O*-GlcNAc modification induced by hyperglycemia competes with phosphorylation, thereby competitively inhibiting eNOS activation and the subsequent NO production. NO is the key mediator of RIC-induced vasodilation and cytoprotection [69]. Second, acute hyperglycemia can directly inhibit Akt phosphorylation. This prevents the effective inhibition of its downstream target glycogen synthase kinase-3β (GSK-3β), consequently promoting the opening of the mitochondrial permeability transition pore (mPTP) and driving cellular apoptosis [70,71]. Notably, this inhibition is partially glucose-dependent. Experiments in classical ischemic preconditioning demonstrate that normalizing blood glucose levels via intensive insulin therapy can restore Akt phosphorylation and reinstate the protective effects of RIC [72], suggesting a similar requirement for glycemic control in RIC therapy. Impairment of the RISK pathway contributes to RIC resistance in the diabetic myocardium. A specific mechanism involves the competitive inhibition of eNOS by excessive O-GlcNAc modification [65]. Although direct evidence for this specific pathway in cerebral ischemia models is currently limited, this cardiovascular evidence provides a critical theoretical foundation for understanding the loss of cerebroprotection. Because diabetes triggers systemic endothelial dysfunction, and given the central role of eNOS-derived NO in both propagating the systemic RIC signal and maintaining the neurovascular unit (NVU) [73], it is highly likely that similar metabolic blockades occur within the cerebrovasculature [74,75]. Therefore, translating these well-defined myocardial resistance mechanisms into the context of diabetic stroke models represents a critical, yet unexplored, frontier for future preclinical research.

However, the scenario is further complicated in the insulin-resistant state characteristic of type 2 diabetes. Chronic hyperinsulinemia compromises the PTEN/Akt/eNOS axis by downregulating microRNA-21, while concurrently activating the MAPK/ET-1 pathway, which aggravates endothelial dysfunction [76]. Additionally, the increased expression of signaling mediators associated with insulin resistance, such as proline-rich tyrosine kinase 2 (PYK2), promotes the inhibitory phosphorylation of eNOS at the tyrosine 657 (Tyr657) site. This counteracts the protective signaling of RIC [77,78,79]. These mechanisms form a complex molecular network that exerts multi-level, multi-targeted inhibition on the RISK pathway in hyperglycemia and insulin resistance. Such a blockade impairs RIC-induced signaling initiation and transmission, thereby inhibiting the entire pathway. This explains why conventional, standardized RIC regimens exhibit attenuated efficacy in diabetic populations. Emerging evidence suggests that extending the therapeutic window, for instance through chronic conditioning over weeks or increasing the number of stimulus cycles, may amplify signal intensity and partially salvage protective efficacy via compensatory mechanisms [62,80]. Therefore, conventional standard RIC stimulation often struggles to overcome the aforementioned pathological resistance in patients with long-standing uncontrolled diabetes and advanced DSPN. Future clinical translation efforts in stroke therapies should focus on developing enhanced intervention strategies. Combining targeted pharmacological interventions, such as p35/CDK5 inhibitors or metabolic modulators, with RIC stimulation could be hypothesized as a potentially effective strategy for restoring endogenous protective pathways in diabetic patients, pending rigorous preclinical validation.

### 3.2. Divergent Regulation of RIC Signal Transmission by Antiplatelet Therapy

Antiplatelet therapy is the cornerstone of secondary prevention for ischemic cardio- and cerebrovascular diseases. Recent studies indicate that platelets function beyond coagulation, serving as critical vehicles for the humoral transmission of RIC-induced protective signals [81]. Thus, pharmacological agents that compromise platelet function may unintentionally interrupt the transmission of RIC protective signaling [82]. Translational studies indicate that platelets play an indispensable role in this transport mechanism. For example, platelets isolated from human volunteers undergoing RIC significantly reduce infarct size when transfused into isolated rat hearts. However, this cardioprotective effect is eliminated when the volunteers are given specific antiplatelet agents before the RIC intervention [82].

Although they act via distinct mechanisms, both aspirin and clopidogrel, the most commonly used clinical antiplatelet agents, markedly impair the transmission of RIC-induced protective signaling. Mechanistic investigations reveal that RIC-induced systemic protection relies on the regulated release of platelet α-granules and dense granules. These granules are rich in serotonin and vascular endothelial growth factor (VEGF), which act as critical mediators for activating vascular endothelial cells and initiating downstream anti-inflammatory and anti-apoptotic programs [83,84]. It has been demonstrated that long-term high-dose aspirin exerts a significant inhibitory effect on thromboxane A2 synthesis, a process that involves the irreversible acetylation of cyclooxygenase-1 (COX-1). This process leads to the significant inhibition of physiological granule release [85,86]. In accordance with this observation, the P2Y12 receptor antagonist clopidogrel has been demonstrated to not only inhibit platelet aggregation but also significantly diminish the generation of EVs laden with protective miRNAs [87]. Notably, the residual platelet activity observed during clopidogrel therapy fails to translate into a protective output. Instead, it is associated with reduced levels of anti-inflammatory cytokines such as interleukin-35 (IL-35) and transforming growth factor-β (TGF-β) [88]. This pharmacological blockade impairs the effective packaging and transport of bioactive mediators of RIC-induced protection, mechanistically explaining why these agents attenuate the clinical benefits of RIC.

Conversely, the novel P2Y12 antagonist ticagrelor has been shown to possess distinctive protective signaling-preserving properties. In contrast to clopidogrel, ticagrelor functions as a direct, reversible P2Y12 antagonist and exhibits unique pleiotropic effects that are independent of P2Y12 inhibition. This preservation is primarily attributable to the inhibition of equilibrative nucleoside transporter 1 (ENT1) on erythrocytes. The aforementioned blockade has been demonstrated to impede the cellular uptake of adenosine, thereby resulting in elevated plasma adenosine levels [89,90,91]. Adenosine, a pivotal humoral mediator of RIC, has been shown to accumulate and directly activate adenosine receptors on cardiomyocytes, thereby initiating protective signaling cascades [91]. Experimental data demonstrate that ticagrelor intrinsically induces a preconditioning-like cardioprotective state, which exerts an additive effect when combined with RIC [82]. Importantly, platelets isolated from volunteers administered ticagrelor retain the capacity to transmit the protective effects of RIC, a finding distinct from the marked blockade observed with aspirin and clopidogrel [82]. This implies that in the design of stroke clinical trials, the therapeutic efficacy of RIC may be masked in patients receiving clopidogrel. Conversely, patients treated with ticagrelor may be better primed to manifest the cerebroprotective benefits of RIC.

The opposing effects of commonly used antiplatelet agents on RIC-mediated protection provide a critical pharmacological explanation for the well-recognized heterogeneity of clinical outcomes in previous RIC randomized trials. It is hypothesized that many prior neutral trials may be partially compromised by high rates of clopidogrel and aspirin use in study cohorts. These agents have been shown to markedly attenuate the humoral transmission of protective signaling. These findings highlight the need for future clinical trial designs to incorporate background antiplatelet regimens as a critical stratification variable. Furthermore, ticagrelor may be the most promising pharmacological adjunct to RIC, thereby maximizing its potential for cardiovascular and cerebrovascular protection.

### 3.3. Anesthesia and Antioxidant Therapies-Induced Interference of RIC-Related Redox Signaling

The pharmacological modulation of redox signaling represents a primary translational barrier to RIC efficacy. As established in the fundamental principles of conditioning, a transient, physiological burst of ROS is an indispensable triggering signal for initiating cerebroprotection. Consequently, any therapeutic agent that prematurely quenches this redox signal will inherently disable the RIC cascade, leading to Type I resistance.

Propofol serves as the most prominent example of this pharmacological interference. As a widely used intravenous anesthetic, propofol is structurally analogous to vitamin E and possesses potent radical-scavenging activity via its phenolic hydroxyl group. Propofol neutralizes the essential ROS burst at the remote trigger site, thereby preventing the activation of downstream survival kinases such as PKC and Akt. This mechanistic interference was definitively validated in preclinical and clinical studies to markedly attenuate RIC’s protective effects [92,93,94,95]. In contrast, volatile anesthetics such as sevoflurane and isoflurane elicit an anesthetic preconditioning effect. These agents directly open mitochondrial ATP-sensitive potassium (mitoKATP) channels, thereby mimicking and potentially synergizing with RIC’s protective mechanisms [96,97,98,99].

Extending this logic beyond anesthesia, antioxidant therapy merits careful scrutiny as a potential inhibitor of RIC efficacy. This antioxidant activity impedes the downstream inhibition of the mitochondrial permeability transition pore (mPTP), thereby disrupting the signaling cascade specifically within the critical induction window of RIC [100,101]. While free-radical scavengers (e.g., edaravone or high-dose Vitamin C) are clinically intended to reduce massive oxidative damage during reperfusion, their administration during the RIC induction window may inadvertently quench the threshold-level signaling ROS burst.

Ultimately, the structural and functional integrity of the RIC protective signaling network is highly vulnerable to the concurrent pharmacological milieu. To overcome these translational barriers, future clinical trials in stroke must incorporate the anesthesia regimen and antioxidant protocols as key stratification variables. Only by identifying and avoiding such pharmacological masking can the genuine therapeutic potential of RIC be realized in diverse clinical settings.

### 3.4. Impact of Aging and Sex on Cerebral Effector Responsiveness

In addition to comorbidities and pharmacological interventions, intrinsic biological characteristics, specifically aging and sex, constitute critical determinants of RIC sensitivity. Given the shared pathophysiological mechanisms of ischemia–reperfusion injury between the heart and the brain, extensive evidence from myocardial models provides critical insights into the effector desensitization observed in the ischemic brain. Aging is widely recognized as a primary risk factor for the refractory nature of RIC protection [102,103,104,105,106]. Senescent organs exhibit not only a diminished tolerance threshold to ischemia–reperfusion injury but also a blunted response to protective stimuli. First, alongside gene-dependent cytosolic processes, mitochondria act as crucial end-effectors of RIC-elicited protective signaling and undergo degenerative changes during aging. Beyond the decline in respiratory chain complex activity and cardiolipin loss, the localization of Connexin 43 (Cx43) at the mitochondrial membrane is significantly reduced [107,108]. Mitochondrial Cx43 acts as a key mediator regulating potassium influx and maintaining mPTP closure. Its deficiency directly disrupts the protective ROS signaling loop triggered by RIC [107,109,110]. Second, extrapolating from classical ischemic conditioning studies, aging precipitates the concurrent inactivation of two core protective pathways, namely the RISK and SAFE pathways [111,112,113]. In the senescent heart, the impaired phosphorylation capacity of Akt and ERK1/2 relieves the inhibition on GSK-3β, thereby promoting mPTP opening [114]. Moreover, STAT3, the hub of the SAFE pathway, exhibits not only reduced expression but also significantly attenuated phosphorylation and mitochondrial translocation (mitoSTAT3) in aged tissues [103,115]. RIC phosphorylates STAT3 at Tyr705 and Ser727 via the JAK-STAT axis. This phosphorylated STAT3 translocates to mitochondria to modulate the respiratory chain and inhibit mPTP opening [116]. Recent investigations reveal that aging-associated downregulation of sirtuin 2 (SIRT2) leads to STAT3 hyperacetylation. This modification not only suppresses its respiratory chain regulatory function but also promotes cellular senescence via the SIRT2-STAT3-CDKN2B axis [117]. Studies in STAT3-deficient mice confirm that the absence of STAT3 results in the near-complete loss of both preconditioning and postconditioning protection [115], a molecular blockade that likely dictates RIC resistance as well. Furthermore, older adults exist in a state of chronic low-grade inflammation. The sustained release of proinflammatory cytokines, such as TNF-α and IL-6, has been demonstrated to potentially interfere with the immunomodulation necessary for RIC through receptor competition or signal disruption [118]. However, this resistance is not absolute. Intensifying the RIC stimulus, for instance, by increasing the number of ischemic cycles, or utilizing hydrogen sulfide (H_2_S) donors to activate Nrf2 and autophagic flux, has been shown to partially reverse RIC resistance in the senescent heart [119,120].

The influence of sex on RIC cerebroprotective efficacy is nuanced, attributable to both the direct actions of sex hormones and complex disparities in molecular signaling [106,121]. Importantly, as established in classical hypoxic/ischemic preconditioning, the therapeutic effectiveness of conditioning heavily depends on an individual’s baseline resistance to the hypoxic factor [122,123]. This biological principle is directly applicable to RIC. Historically, it was postulated that the presence of endogenous estrogen equips premenopausal females with a higher capacity for ischemic tolerance. In some experimental models, the activation of estrogen receptors (ER) upregulates eNOS expression and enhances mitochondrial function. This theoretical baseline preconditioned state establishes a higher intrinsic resistance to hypoxia, which may create a biological ceiling effect, rendering standard RIC protocols unlikely to provide further therapeutic benefits [124].

The clinical translation of this concept is highly controversial, reflecting the complex biological paradox of estrogen in stroke pathophysiology. Clinical evidence indicates that the impact of endogenous estrogen on stroke outcomes may be negligible in certain premenopausal cohorts. More importantly, elevated estrogen concentrations or exogenous hormone replacement therapies have been shown to paradoxically increase thrombotic stroke risk and aggravate overall outcomes [125,126]. Consequently, the traditional assumption that estrogen acts as a universally beneficial agent following stroke remains heavily debated.

In addition, the remote, systemic transfer of cerebroprotection induced by RIC relies on the transmission of humoral factors. A translational study revealed that while plasma from RIC-treated male volunteers transferred protection to isolated rat hearts, plasma from female volunteers failed to induce a similar effect. This points to a sex-specific difference in the generation or release of critical hydrophobic protective molecules or exosomal miRNAs, thereby limiting the distal efficacy of RIC in females [106]. This biological variance has been corroborated in clinical trials. In the RICA trial targeting chronic ischemia, RIC reduces the risk of recurrent stroke in men but proved ineffective in women [121]. This failure likely reflects dual constraints in the context of chronic prevention, specifically a heavier comorbidity burden, such as microangiopathy, and molecular resistance characterized by sirtuin (SIRT) deficiency. In contrast, under the intense stress of acute ischemia, as evidenced by the RICAMIS trial, RIC successfully improved prognosis in female patients [127]. We propose that this discrepancy reflects differences in baseline ischemic tolerance. Under chronic ischemic conditions, estrogen provides endogenous protection in premenopausal women, establishing a higher baseline ischemic tolerance. This may create a biological ceiling effect that limits the additional efficacy of standard RIC protocols. Conversely, during acute ischemic events, the relative reduction in baseline ischemic tolerance allows RIC to exert a measurable protective effect.

Furthermore, despite the debate surrounding estrogen-mediated direct protective effects, the profound endocrinological shift and loss of cyclic hormone regulation during menopause undeniably alter the baseline neurovascular network. This transition to a postmenopausal state results in a high-threshold phenotype comparable to that of elderly men, which aligns with Type III RIC resistance. This sex- and menopause-related difference implies that females may harbor a higher activation threshold for RIC. In chronic adaptive settings, standard RIC protocols are insufficient to overcome this blockade of RIC protective signaling. Under conditions of acute ischemic stroke or severe ischemic injury, the intense endogenous stimulation may temporarily override these biological limitations.

### 3.5. The Role of Vascular Health and Smoking in RIC Signal Conduction

Hemodynamic status and lifestyle factors also represent key limiting factors for RIC cerebroprotective efficacy. The effective delivery of RIC humoral mediators to the ischemic penumbra is strictly contingent upon the patency of the systemic circulation [128]. Severe arterial occlusion with poor collateral flow prevents protective factors from reaching the cerebral target tissues. Studies indicate that RIC acts by promoting collateral vessel recruitment and preventing the collapse of leptomeningeal collaterals [129,130]. A physiological paradox thus arises, as RIC depends on the same collateral vessels it intends to improve. Consequently, preclinical and preliminary clinical data suggest that RIC cerebroprotective efficacy is significantly attenuated in patients with severely compromised collateral circulation [131,132]. To address this complexity, the ongoing RICAS study uses Digital Subtraction Angiography (DSA) as the gold standard to clarify how RIC improves collateral perfusion and its dependence on baseline vascular status [133].

The impact of smoking on RIC effectiveness remains controversial, reflecting complex underlying pathophysiological mechanisms. Subgroup analysis of the RICAMIS trial revealed that RIC conferred significant prognostic benefits in non-smokers, whereas it proved ineffective in current smokers [134]. Mechanistically, chronic smoking induces endothelial dysfunction and systemic oxidative stress, reducing NO bioavailability. These pathological changes likely compromise the endothelial redox signaling pathways upon which RIC relies [135,136]. Nicotine has also been shown to abolish the protective effects of local ischemic postconditioning [137], indicating a universal vulnerability of conditioning networks to cigarette smoke. Conversely, clinical studies involving percutaneous coronary intervention (PCI) indicate that smokers exhibit a more significant reduction in myocardial injury following RIC compared to non-smokers, a phenomenon termed the Smoker’s Paradox [138]. This paradox reflects differences in patient baseline characteristics and disease acuity. In elective PCI, smokers are generally younger with fewer comorbidities and a higher thrombotic burden; here, RIC may reduce injury by inhibiting platelet activation. Conversely, in acute ischemic stroke, chronic smoking causes systemic oxidative stress, endothelial dysfunction, and impaired collateral circulation. These pathological changes directly disrupt the initiation and systemic transmission of protective signals, attenuating the efficacy of RIC. Additionally, smoking induces metabolic enzymes, such as CYP1A2, which alter the pharmacokinetics of cardiovascular drugs and serve as a confounding factor. However, in the setting of acute ischemic stroke, most clinical evidence supports that chronic smoking impairs RIC cerebroprotective efficacy and is associated with RIC resistance, which is distinct from the paradoxical benefit observed in elective PCI settings. This discrepancy is likely driven by disease acuity, baseline endothelial function, and thrombotic burden differences between acute and chronic ischemic scenarios.

Similarly, other leading vascular comorbidities may also compromise this endothelial conduit. For instance, hypertension creates a chronic systemic pro-inflammatory state [139] and widespread oxidative stress, with the potential to impair endothelial cells, which serve as major elements of the described signaling cascades. Given that the systemic propagation of RIC relies on vascular reactivity and endothelial-derived NO, hypertensive vascular remodeling might act as a barrier to this transmission. While direct clinical evidence in stroke cohorts remains limited, we propose a working hypothesis that hypertension-induced endothelial dysfunction could represent an additional factor driving Type II resistance, warranting targeted investigation in downstream clinical trials.

## 4. Phenotype-Based Stratification and Precision RIC Strategies

**Figure 2:**
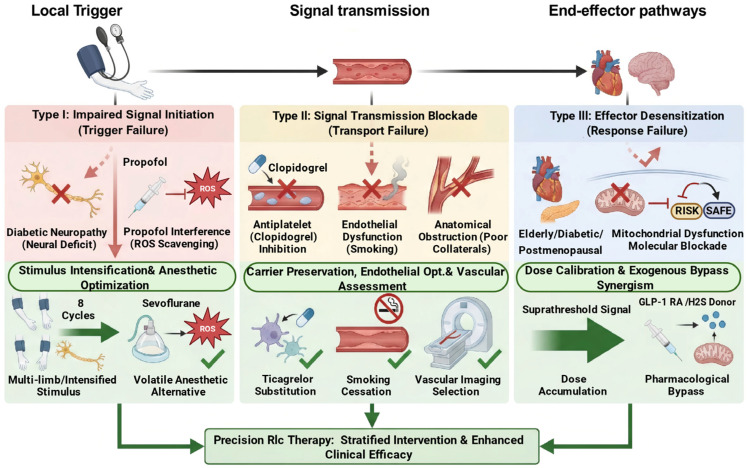
Mechanistic classification of RIC resistance. Pathophysiological factors limiting the clinical efficacy of RIC are categorized into three mechanism-driven phenotypes. Type I (Impaired signal initiation) represents a failure to generate the initial protective trigger, driven by afferent neural deficits (e.g., diabetic neuropathy) or iatrogenic ROS scavenging (e.g., propofol). Restoring initiation requires stimulus intensification or anesthetic optimization. Type II (Signal transmission blockade) occurs when the systemic signal is attenuated by humoral carrier inhibition (e.g., clopidogrel), smoking-induced endothelial dysfunction, or poor collateral circulation. Interventions focus on carrier preservation, smoking cessation, and vascular imaging-based stratification. Type III (Effector desensitization) involves target organ unresponsiveness. This phenotype dominates in high-threshold populations such as the elderly, diabetics, and postmenopausal women, where metabolic stress and estrogen withdrawal precipitate mitochondrial dysfunction and block endogenous survival kinase pathways (RISK/SAFE). Overcoming this desensitization necessitates dose calibration for suprathreshold signaling or exogenous pharmacological bypasses (e.g., glucagon-like peptide-1 (GLP-1) receptor agonists, hydrogen sulfide (H_2_S) donors) to directly activate downstream targets.

Addressing the challenge of translating preclinical RIC cerebroprotective efficacy into clinical practice requires a targeted approach. To address this translational barrier, future preclinical and clinical research should move from empirical treatment to a mechanism-based precision medicine approach. By systematically analyzing the pathophysiological barriers of RIC resistance, we classify it into three mechanism-driven phenotypes (Figure 2): Type I (Impaired signal initiation), Type II (Signal transmission blockade), and Type III (Effector desensitization). This classification lays a theoretical foundation for transforming RIC from a uniform therapy to a stratified intervention. The core of precision RIC therapy is to translate complex pathophysiological mechanisms into clinically actionable eligibility criteria and stratification strategies, while rigorously assessing the efficacy of protective signal transmission in both clinical practice and trial design.

It is noteworthy that patients rarely present with a single risk factor for RIC resistance. More commonly, a combination of overlapping factors, including advanced age, diabetes, and concurrent antiplatelet therapy, exerts synergistic inhibitory effects on multiple nodes of the RIC-induced signaling cascade. This results in more severe, and even complete, RIC resistance that cannot be recapitulated or fully explained by single-factor preclinical or clinical studies.

### 4.1. Type I Resistance: Targeting Impaired Signal Initiation

Type I resistance is defined as a failure to generate the initial protective signal, a phenotype frequently observed in patients with diabetic neuropathy or those undergoing propofol anesthesia. Interventions for this phenotype either compensate for neural deficits via intensified stimulation or reverse inhibitory conditions with pharmacological approaches.

#### 4.1.1. Stimulus Intensification

Diabetic peripheral nerve injury disrupts the afferent limb of the reflex arc, making standard-intensity RIC stimuli unable to elicit effective neural impulses. Given that an intact neural reflex arc is indispensable for RIC cerebroprotective efficacy, patients with severe distal symmetric polyneuropathy require stimulus intensification instead of the standard four-cycle protocol. Specific measures include simultaneous multi-limb ischemia or increasing ischemia/reperfusion cycles to eight. Expanding the ischemic area recruits more residual functional nerve fibers, compensating for conduction deficits and initiating the protective reflex.

#### 4.1.2. Anesthetic Regimen Optimization

Iatrogenic drug interference is another major cause of impaired signal initiation. Propofol scavenges ROS, blunting the redox signaling necessary for RIC initiation. It is hypothesized that clinicians could preoperatively identify at-risk populations and potentially prioritize volatile anesthetics as alternatives, pending validation in preclinical stroke models. For example, sevoflurane not only preserves ROS signaling but also mimics preconditioning by opening ATP-sensitive potassium channels, counteracting propofol-induced blockade of RIC protective signal initiation.

### 4.2. Type II Resistance: Targeting Signal Transmission Blockade

Type II resistance occurs when the RIC-mediated protective signal is successfully initiated but attenuated or interrupted during transmission, caused by humoral carrier inhibition, endothelial dysfunction or anatomical obstruction.

#### 4.2.1. Antiplatelet Therapy Optimization

Antiplatelet therapy is a key iatrogenic factor disrupting humoral transmission. Clopidogrel irreversibly inhibits P2Y12 receptors, blocking platelet granule release and impairing a critical humoral transport mechanism. In contrast, ticagrelor offers a unique therapeutic advantage. For acute ischemic conditions requiring antiplatelet therapy, drug substitution with ticagrelor could be explored as a potential strategy, where clinically appropriate and in accordance with current guidelines. Ticagrelor preserves platelet-mediated signal transmission and enhances RIC cerebroprotective efficacy by inhibiting ENT1-mediated adenosine uptake, thereby elevating plasma adenosine levels.

#### 4.2.2. Endothelial Function Optimization

In acute ischemic scenarios, smoking is a significant risk factor for RIC transmission failure, although its impact in elective procedures remains complex. Chronic smoking induces severe oxidative stress, leading to endothelial dysfunction that compromises the vascular endothelium’s role in transmitting and amplifying humoral signals such as nitric oxide and microRNAs. Future clinical trials should incorporate smoking status as a stratification variable. For patients undergoing elective surgery where baseline endothelial dysfunction is the primary translational barrier, a strict preoperative smoking cessation period is recommended to partially restore endothelial function, while carefully accounting for potential confounders such as the smoker’s paradox. Additionally, heavy smokers should be analyzed as a separate subgroup to avoid confounding results from endothelial transmission barriers that mask the true efficacy of RIC in non-smoking populations.

#### 4.2.3. Vascular Condition Assessment

In pathological conditions such as acute ischemic stroke, the delivery of humoral mediators to the ischemic penumbra relies heavily on residual blood flow from collateral circulation. Poor collateral circulation constitutes a physical barrier to signal transmission. Thus, vascular imaging techniques (e.g., CTA, DSA) should be integrated into routine pre-enrollment screening to assess collateral integrity. In clinical practice, hemodynamic-based patient stratification should be implemented. RIC may be more beneficial for patients with adequate collaterals, while those with severe collateral deficiency, where humoral factors cannot reach the ischemic penumbra, might be excluded from standard RIC protocols to avoid futile treatment.

### 4.3. Type III Resistance: Targeting Effector Desensitization

Type III resistance mainly affects the elderly, patients with chronic hyperglycemia and postmenopausal women. These populations exhibit target organ mitochondrial dysfunction and molecular blockade of survival kinase pathways, including RISK and SAFE, resulting in marked effector desensitization to RIC. Therapeutic strategies should therefore break through this desensitization threshold by either enhancing physical stimulus intensity or pharmacologically activating endogenous protective pathways.

#### 4.3.1. Dose Calibration and Signal Amplification

In the senescent neurovascular unit, diabetic target organs and post-estrogen withdrawal states, standard RIC stimulation is insufficient to overcome intracellular molecular blockades such as SIRT2 downregulation or *O*-GlcNAc accumulation. Clinical trials should implement multidimensional stratification accounting for age–sex interactions. Postmenopausal women display desensitization similar to the elderly due to lost estrogen-mediated mitochondrial protection, and thus should be classified with elderly males as a high-threshold phenotype. For these populations, stimulus intensification is required, which must encompass a comprehensive calibration of the physical RIC parameters. Protocol customization must extend beyond cycle frequency to include individualized pressure and duration. For instance, in elderly patients with severe arterial stiffness or diabetic patients with hypertension, a fixed 200 mmHg cuff pressure (without adjusting for individual baseline blood pressure) may fail to induce complete arterial occlusion, resulting in an insufficient ischemic trigger. Individualizing target inflation pressure strictly to 50 mmHg above the patient’s baseline systolic blood pressure and extending ischemic phase duration ensures sufficient local metabolic shift. Unlike Type I resistance, this strategy relies on dose accumulation to generate a suprathreshold protective signaling cascade, utilizing optimized physical parameters to elevate peak concentrations of effector molecules like adenosine and bradykinin, thereby overcoming receptor desensitization in ischemic brain.

#### 4.3.2. Pharmacological Augmentation

In refractory cases with severe endogenous signaling impairment, such as elderly diabetic patients, physical stimulus intensification alone may be inadequate. A pharmacological bypass strategy could be investigated by combining RIC with exogenous signal enhancers. Clinically, agents like GLP-1 receptor agonists or hydrogen sulfide donors can directly activate downstream RISK/SAFE pathways or stabilize mitochondrial function. This approach is hypothesized to bypass compromised upstream signal cascades, restoring RIC-mediated protective efficacy in cerebral tissue.

## 5. Conclusions and Future Perspectives

**Figure 3:**
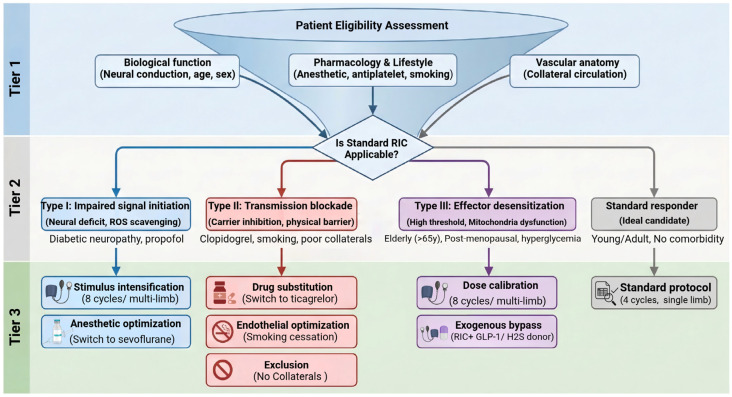
Mechanism-based precision treatment algorithm for remote ischemic conditioning. A tiered clinical decision matrix translating pathophysiological resistance into customized therapeutic strategies. Tier 1 (Multidimensional screening) evaluates baseline biological function, pharmacological/lifestyle history, and vascular anatomy to determine initial patient eligibility. Tier 2 (Phenotypic profiling) stratifies patients into ideal candidates for standard protocols or assigns them to specific resistance phenotypes: Type I (Impaired signal initiation), Type II (Transmission blockade), and Type III (Effector desensitization). Tier 3 (Targeted intervention) deploys phenotype-guided countermeasures. High-threshold phenotypes (Types I and III) are managed via stimulus intensification, dose calibration, or exogenous bypass (e.g., GLP-1 receptor agonists), whereas iatrogenic and physical barriers (Types I and II) necessitate anesthetic optimization, drug substitution (e.g., ticagrelor), or clinical exclusion to prevent futile care.

RIC clinical translation in stroke cerebroprotection is transitioning towards mechanism-based precision strategies. By integrating the pathophysiological mechanisms of RIC resistance with clinically actionable screening criteria and individualized interventions, we have developed a phenotype-based conceptual clinical framework for precision RIC therapy (Figure 3). This framework consists of three key steps. First, multidimensional screening establishes a standardized evaluation checklist covering three domains: biological function (neural conduction, age, menopausal status), lifestyle and pharmacological history (smoking, antiplatelet use), and vascular condition (collateral circulation integrity). Second, phenotypic profiling uses screening results to classify patients into Type I, II or III RIC resistance, clarifying the core pathological mechanism underlying impaired RIC cerebroprotective efficacy. Third, targeted intervention deploys phenotype-specific strategies, stimulus intensification for Type I and III high-threshold phenotypes; regimen optimization and endothelial restoration to rescue signal transmission for Type I and II; and pharmacological augmentation to augment endogenous protection for refractory Type III.

### 5.1. Limitations of Current RIC Resistance Research

Several limitations currently impede the clinical management of RIC resistance. Primarily, the absence of validated circulating biomarkers or unified diagnostic criteria hinders systematic patient stratification. Furthermore, most preclinical models rely on young, healthy, single-etiologic animals, failing to recapitulate the complex polypharmacy and multi-comorbidity profiles of real-world clinical stroke populations. Therefore, while modeling the entire spectrum of clinical complexities remains challenging, a pragmatic intermediate step is to utilize preclinical models incorporating single or paired comorbid factors. As successfully demonstrated in cardioprotection research, this approach provides a highly valuable platform to validate the targeted interventions proposed for each resistance phenotype. Crucially, as discussed in this review, a significant portion of the current mechanistic evidence regarding pharmacological barriers (e.g., antiplatelet and anesthetic interference) is derived from myocardial infarction models. While the heart and brain share fundamental ischemia–reperfusion injury pathways, the precise impact of these specific factors on the neurovascular unit requires rigorous validation in dedicated stroke models. Consequently, the synergistic inhibitory effects of overlapping risk factors remain poorly understood. Finally, existing mechanistic evidence predominantly derives from single-center, small-sample studies, lacking validation from large-scale, multicenter randomized controlled trials (RCTs).

### 5.2. Future Perspectives

To synthesize the mechanism-driven phenotypes discussed throughout this review and provide actionable guidance for the field, Table 1 summarizes the primary pathophysiological barriers, targeted clinical optimization strategies, and specific recommendations for future clinical trial design.

Future trials should move beyond testing standard RIC protocols in unstratified cohorts. To achieve successful clinical translation, trial designs must integrate the precision-driven enrichment strategies outlined in Table 1, with a specific focus on acute ischemic stroke. This involves rigorous control of background medications such as antiplatelets and anesthetics in the acute setting and the utilization of advanced neuroimaging to prioritize patients with favorable collateral flow profiles, as these profiles appear to be critical determinants of RIC cerebroprotective efficacy. Stratified randomized controlled trials comparing standard versus intensified RIC in high-risk subgroups are essential. Furthermore, given the profound impact of the aging brain on ischemic vulnerability, the investigation of exogenous signal enhancers to overcome Type III resistance potentially offers a viable path for the geriatric stroke population. Ultimately, the discovery of robust clinical biomarkers may be pivotal for establishing a diagnostic framework for phenotypic stratification. Through rigorous and mechanism-driven trial designs, RIC could advance from a generalized empirical procedure toward a standardized and precision cerebroprotective strategy for acute ischemic stroke.

## Figures and Tables

**Table 1:** Mechanism-driven phenotypes of RIC resistance, targeted clinical optimization strategies, and recommendations for future clinical trial design.

RIC Resistance Phenotype	Primary Pathophysiological/Pharmacological Barriers	Targeted Clinical Optimization Strategies	Recommendations for Future Trial Design
**Type I**: Impaired signal initiation	• **Diabetic Neuropathy (DSPN):** Structural/molecular impairment of afferent neural pathways.	• **Stimulus Intensification**: Increase ischemia cycles (e.g., 8 cycles) or apply multi-limb conditioning.	• **Subgroup Allocation**: Patients with severe DSPN may require separate intensive protocol arms.
	• **Propofol Anesthesia**: Iatrogenic scavenging of the required ROS burst.	• **Anesthetic Optimization**: Prioritize volatile anesthetics (e.g., sevoflurane).	• **Mandatory Stratification**: Stratify cohorts strictly by background anesthetic regimens.
**Type II**: Signal transmission blockade	• **Antiplatelet Therapy**: Clopidogrel blocks protective humoral (granule) release.	• **Drug Substitution**: Substitute with ticagrelor where appropriate to preserve adenosine signaling.	• **Background Medication Control**: Antiplatelet regimens must be a primary stratification variable.
	• **Endothelial Dysfunction**: Hypertension and smoking impair NO-dependent propagation.	• **Endothelial Restoration**: Strict preoperative smoking cessation for elective cases.	• **Imaging Integration**: Integrate vascular imaging (CTA/DSA) as routine pre-enrollment screening.
	• **Anatomical Obstruction**: Poor collateral flow limits signal delivery.	• **Patient Selection**: Exclude patients with severe collateral deficiency from standard protocols.	
**Type III**: Effector desensitization	• **Senescence**: Degeneration of Cx43 and functional decline of RISK/SAFE pathways.	• **Dose Calibration**: Individualize inflation pressure (baseline systolic + 50 mmHg) and extend ischemic duration.	• **Baseline Biological Profiling**: Implement strict age–sex interaction analyses.
	• **The Estrogen Paradox**: Endocrine shifts alter baseline tolerance and blunt responsiveness.	• **Pharmacological Augmentation**: Combine RIC with exogenous enhancers (e.g., GLP-1 agonists).	• **Dose-Finding Studies**: Design adaptive trials comparing standard vs. intensified RIC in high-threshold subgroups.
	• **Chronic Hyperglycemia**: O-GlcNAc accumulation competitively inhibits signaling.		

## Data Availability

No new data were created or analyzed in this study.
